# Scalable Microgravity Simulator Used for Long-Term Musculoskeletal Cells and Tissue Engineering

**DOI:** 10.3390/ijms21238908

**Published:** 2020-11-24

**Authors:** Alessandra Cazzaniga, Fabian Ille, Simon Wuest, Carsten Haack, Adrian Koller, Christina Giger-Lange, Monica Zocchi, Marcel Egli, Sara Castiglioni, Jeanette A. Maier

**Affiliations:** 1 Department of Biomedical and Clinical Sciences L. Sacco, Università di Milano, 20157 Milan, Italy; alessandra.cazzaniga@unimi.it (A.C.); monica.zocchi@unimi.it (M.Z.); jeanette.maier@unimi.it (J.A.M.); 2 Space Biology Group, Institute of Medical Engineering, Lucerne University of Applied Sciences and Arts, 6002 Lucerne, Switzerland; fabian.ille@hslu.ch (F.I.); simon.wueest@hslu.ch (S.W.); christina.giger@hslu.ch (C.G.-L.); marcel.egli@hslu.ch (M.E.); 3 Institute of Mechanical Engineering and Energy Technology, Lucerne University of Applied Sciences and Arts, 6002 Lucerne, Switzerland; carsten.haack@hslu.ch (C.H.); adrian.koller@hslu.ch (A.K.); 4 Interdisciplinary Centre for Nanostructured Materials and Interfaces (CIMaINa), Università di Milano, 20133 Milan, Italy

**Keywords:** simulated microgravity, bone marrow stem cells, myoblasts

## Abstract

We introduce a new benchtop microgravity simulator (MGS) that is scalable and easy to use. Its working principle is similar to that of random positioning machines (RPM), commonly used in research laboratories and regarded as one of the gold standards for simulating microgravity. The improvement of the MGS concerns mainly the algorithms controlling the movements of the samples and the design that, for the first time, guarantees equal treatment of all the culture flasks undergoing simulated microgravity. Qualification and validation tests of the new device were conducted with human bone marrow stem cells (bMSC) and mouse skeletal muscle myoblasts (C2C12). bMSC were cultured for 4 days on the MGS and the RPM in parallel. In the presence of osteogenic medium, an overexpression of osteogenic markers was detected in the samples from both devices. Similarly, C2C12 cells were maintained for 4 days on the MGS and the rotating wall vessel (RWV) device, another widely used microgravity simulator. Significant downregulation of myogenesis markers was observed in gravitationally unloaded cells. Therefore, similar results can be obtained regardless of the used simulated microgravity devices, namely MGS, RPM, or RWV. The newly developed MGS device thus offers easy and reliable long-term cell culture possibilities under simulated microgravity conditions. Currently, upgrades are in progress to allow real-time monitoring of the culture media and liquids exchange while running. This is of particular interest for long-term cultivation, needed for tissue engineering applications. Tissue grown under real or simulated microgravity has specific features, such as growth in three-dimensions (3D). Growth in weightlessness conditions fosters mechanical, structural, and chemical interactions between cells and the extracellular matrix in any direction.

## 1. Introduction

Commonly used static two-dimensional (2D) cell culture techniques have contributed substantially to many breakthroughs in cell biology during the last decades. More recently, devices that introduce the third-dimension (3D) have offered novel insights. 3D dynamic bioreactors overcome diffusional limitations of static culture, thus improving the quality of different types of engineered tissues and optimizing cell proliferation and differentiation, e.g., for regenerative medicine. Some of these 3D bioreactors have been used to simulate microgravity. Indeed, weightlessness offers advantages in the area of tissue engineering [1], as sedimentation and buoyancy are negligible, facilitating the accurate observation of alterations in biological systems. Consequently, simulated microgravity has been proposed as a challenging opportunity to grow cells as well as to generate organoids, spheroids, or tissues with and without scaffolds, which can be used for drug testing, tissue engineering, and regenerative medicine [2,3,4]. 

Among the bioreactors, the rotating wall vessel (RWV), a suspension culture system that permits the cultivation of cells under low physiological fluid shear conditions, has been widely utilized [5,6]. The RWV requires to grow adherent cells on microcarrier beads so that they rotate at the same speed of the vessel wall around a horizontal axis, a condition that produces a vector-averaged gravity comparable with that of the near-earth free-fall orbit [7].

The random positioning machine (RPM), also referred to as 3D-clinostat, mimics microgravity conditions by rotating samples around two axes through which the gravitational acceleration on the samples changes direction ceaselessly. Averaged over time, the residual acceleration at the intersection of the two rotation axes where the samples are placed reaches near-zero mathematically [8]. The RPM has been extensively used to study the effects of microgravity on biological or physical processes [9,10]. To culture cells on the RPM, they have to be grown in cell chambers or in flasks entirely filled with media to minimize shear stress [11]. It is noteworthy that experiments performed in the RVW and RPM yield similar results and corroborate data reported in real microgravity [10,12]. Moreover, both the RWV and the RPM have proven to be elegant tools for tissue engineering [13].

The major drawback of the RPM technique, however, is the particular movement used to generate the microgravity environment. This movement introduces supplementary forces that may trigger non-specific cellular responses. We have been studying the appearance of such forces and their impact on undesired cellular effects by applying mathematical modeling extensively. In this study, we were able to define particular rules to be followed to avoid additional cell responses [14]. 

Here, we describe a novel microgravity simulator (MGS) that evolved from the well-known RPM principle. With our new MGS, we resolve some essential drawbacks of the classical RPM, thus improving substantially the overall quality of simulated microgravity cultivation. We introduce a new control algorithm and new measures for the rotating systems that generate low gravity conditions. We then determined the effects of culture in the MGS vs. RVW or RPM on musculoskeletal cells, as real microgravity markedly affects the musculoskeletal systems. Rather than using differentiated cells, we focused on progenitor cells. In particular, we concentrated on the osteogenic differentiation of human bone marrow stem cells (bMSC) and the myogenic differentiation of mouse myoblasts. We conclude that culture on the MGS yields results that are similar to those obtained using the RWV or the RPM. Thus, our new MGS can be used for cell biological experiments under simulated microgravity conditions, which can also be specified as a mechanically unloaded environment.

## 2. Results

### 2.1. Description of the MGS

The main goals and requirements for the novel MGS design are summarized by the following technical and usability aspects:

1. Increased availability of a simulated microgravity environment by a simple desktop machine
2. Down-sizing for the use in typical lab environments (e.g., fitting into CO_2_ cell incubators)
3. Reproducible and optimized motion control (uniformity and nullification)
4. Possibility to use and analyze several samples in parallel
5. Scalable design (fulfilling the needs of users)
6. Identical treatment of all samples (equality)
7. Interface commonly used in cell cultures
8. Simplified LabVIEW interface that allows an easy control of all the MGS functions.

**Figure 1:**
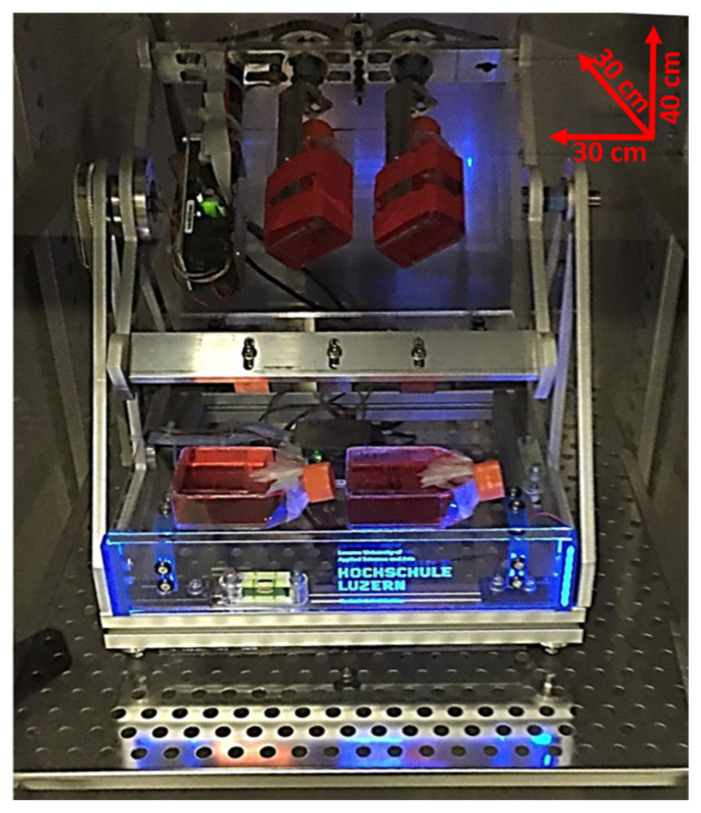
Image of the microgravity simulator (MGS) hosted in a CO_2_ incubator. The dimensions are reported. The MGS is connected to a PC that runs a particular software based on a two-level software architecture approach to enable a wide range of applications: A first-level offline motion simulation for parameter identification and optimization for different targets and ranges of microgravity conditions, and a second-level drive control software using these optimized parameters in the microgravity simulator (MGS-hardware).

Thanks to its compact design, the MGS can be accommodated in a commercially available CO_2_ incubator (Figure 1). 

**Figure 2:**
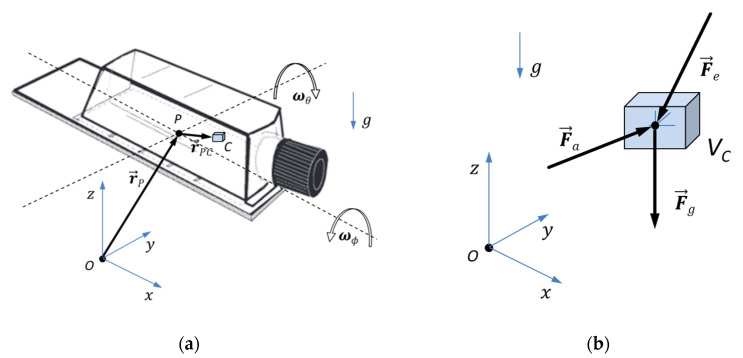
(**a**) Position vectors *r* in a typical rotating set-up with angular velocities ω; (**b**) Forces acting on a single cell.

The set-up of the MGS is focused on obtaining equal treatment of all samples of one batch (Figure 2a). Their movement follows a particular motion and trajectory pattern that was calculated by using MATLAB/LabVIEW (MathWorks) to ensure adequate nullification of the gravity vector at a range of 10^−3^ g. A smoothed path control during reversals and velocity changes results in the minimal introduction of shear forces and structural vibration of the machine (reducing high-frequency interference effects). 

The forces acting on a single cell on the operating MGS or the respective control volume *V_c_* in a rotating set-up like the one used to simulate microgravity are depicted in Figure 2b (mass *m* = *ρ* × *V_c_*, with density *ρ*), where *F_a_* = *m* × *a* gives the apparent force, due to the changing motion and acceleration of the flask (the total acceleration a is given by kinematics of cell positions), *F_g_* = *m* × *g* gives the gravity force (with gravitational acceleration *g* = 9.80665 m/s^2^, changing its relative direction during operation, in a cell reference frame), and *F_e_* other external forces, such as shear and magnetic forces, are neglected because they are of minor influence. 

The principle idea behind all rotating microgravity devices is shown in Figure 2a,b, where *C* represents an arbitrary position of a single cell with a control volume *V_c_*. For any motion or rotation, the position of point *C* is described by the time-dependent position vector:

*r_c_*(*t*) = *r_p_*(*t*) + *r_pc_*(*t*)

The respective velocities, absolute or relative accelerations for the cell *C* are given by time derivatives d/dt and d^2^/dt^2^ of this position vector *r_c_*(*t*). The equilibrium equation for the control volume gives the respective forces (which can also include shear forces from flow velocities). Simulations can be run in advance, based on the above defined mathematical relations that include smoothed path control during reversals and velocity changes (including jerk limitations *da*/*dt*) to illustrate the quality of the simulated microgravity and the evolving forces. More details about the MGS, as well as the description of the algorithm used to simulate microgravity, are available in the Methods section.

Besides validating the MGS mathematically, biological tests were conducted to compare the cellular behaviour on the MGS and on commonly used microgravity simulators.

### 2.2. bMSC: Effects of Culture in MGS vs. RPM

**Figure 3:**
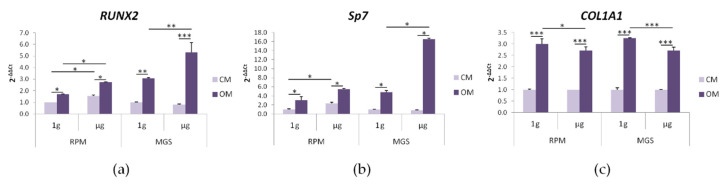
bMSC were cultured in the RPM or in the MGS as well as in 1 g-ground control for 4 days. Three different experiments were performed. RT-PCR was performed three times in triplicate on RNA extracted using primers designed on *RUNX2* (**a**), *Sp7* (**b**), and *COL1A1* (**c**) sequences. * *p* ˂ 0.05; ** *p* ˂ 0.01; *** *p* ˂ 0.001.

Bone loss due to weightlessness represents a significant health issue and, while there is clear evidence of an altered behaviour of osteoblasts and osteoclasts, less is known about the effects of microgravity on bMSC. Therefore, we compared the expression of osteogenic markers in human bMSC cultured in their culture medium (CM) or the osteogenic medium (OM) containing vitamin D for 4 days in the RPM or the MGS. Cells in static 1g-ground control posed at the base of each bioreactor were used as controls (1g). The expression of *RUNX2*, *Sp7*, and *COL1A1* was investigated by RT-PCR. *RUNX2* (Runt-related transcription factor 2) encodes for a transcription factor that acts as the master regulator of osteogenesis. *Sp7* encodes for Osterix, another transcription factor required for osteoblast differentiation. *COL1A1* encodes for collagen type 1, which is the most abundant extracellular protein in bone [15]. As shown in Figure 3, OM induces a significant increase of all the transcripts both in 1g-ground controls and in cells in the RPM and MGS. The induction of *RUNX2* and *Sp7* was significantly higher in bMSC cultured in OM in the MGS than in the RPM (Figure 3a,b), while the expression of *COL1A1* was similar (Figure 3c). As previously reported [16], it was sufficient to culture the cells in their CM in the RPM for 4 days to upregulate *RUNX2* and *Sp7*, even in the absence of the osteogenic cocktail. Interestingly, this did not happen in the MGS, suggesting that stimuli other than gravitational unloading might be implicated in modulating bMSC behaviour in the RPM.

**Figure 4:**
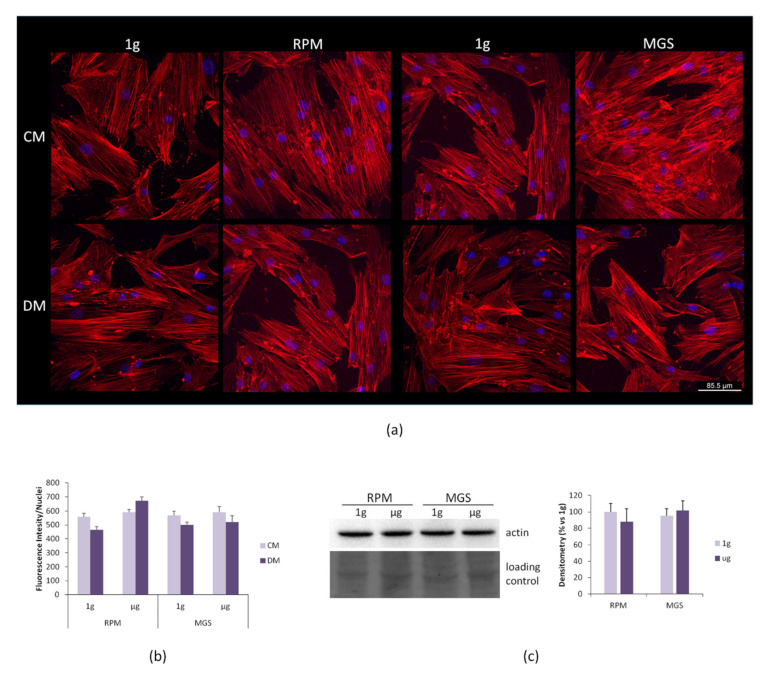
bMSC were cultured in the RPM or in the MGS as well as in 1 g-ground control for 4 days. (**a**) After fixation, the cells were stained with rhodamine labeled phalloidin and 4′,6-diamidine-2′-phenylindole dihydrochloride (DAPI). (**b**) Phalloidin fluorescence was quantified by ImageJ. (**c**) Western blot using antibodies against β-actin was performed on cell extracts of bMSC cultured in 1g or simulated microgravity in standard culture medium (CM). The lower panel shows the loading control. Densitometry was performed using ImageJ.

bMSC were then cultured in the RPM or in the MGS for 4 days, and cell morphology was examined by confocal microscopy after staining with phalloidin to detect actin filaments of the cytoskeleton and DAPI to visualize the nuclei. No significant differences in cell shape or in the distribution and organization of the actin fibers were observed in the cells cultured in the two devices in CM or OM (Figure 4a). The actin fibers were quantified, and no significant differences emerged (Figure 4b). We then evaluated the total amounts of actin in bMSC cultured for 4 days in simulated microgravity in CM vs. their controls in 1 g by western blot and found no significant modulation of actin levels (Figure 4c). 

### 2.3. C2C12 Myoblasts: Effects of Culture in MGS vs. RWV

**Figure 5:**
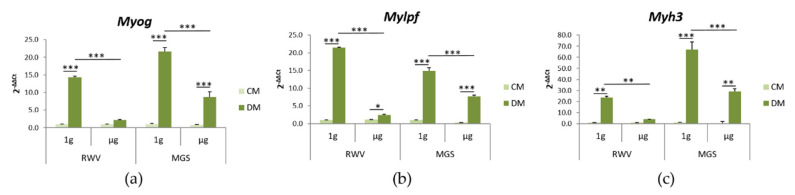
Mouse skeletal C2C12 myoblasts were cultured both in the RWV and in the MGS or in 1 g-ground control for 4 days. RT-PCR was performed three times in triplicate on RNA extracted using primers designed on *Myog* (**a**), *Mylpf* (**b**), and *Myh3* (**c**) sequences. * *p* ˂ 0.05; ** *p* ˂ 0.01; *** *p* ˂ 0.001.

Skeletal muscle volume and peak power significantly decrease after prolonged exposure to microgravity [17,18]. This observation is explained by the evidence that myogenesis is inhibited in simulated microgravity [19,20]. Here, we compared the effects of microgravity generated by MGS with the RWV. We utilized mouse skeletal C2C12 myoblasts. C2C12 were cultured for 4 days in the two bioreactors containing their standard culture medium (CM) and in the myogenic medium (differentiation medium, DM) containing 2% horse serum. C2C12 maintained on the base of each bioreactor were used as 1 g-ground control. By RT-PCR, we analyzed the expression of some markers involved in myogenic differentiation, i.e., *Myog*, *Mylpf*, and *Myh3*. *Myog* encodes myogenin, the transcription factor that coordinates myogenesis [21]. *Mylpf* encodes for the myosin light chain 2, while *Myh3* encodes for myosin heavy chain 3 (MHC), both involved in the regulation of contraction [22]. As shown in Figure 5, the DM induces a marked upregulation of all the transcripts in the 1g-ground samples. In agreement with previous studies using various bioreactors to simulate microgravity [23], culture in the MGS or in the RWV in DM significantly downregulated *Myog*, *Mylpf*, and *Myh3* transcript. 

**Figure 6:**
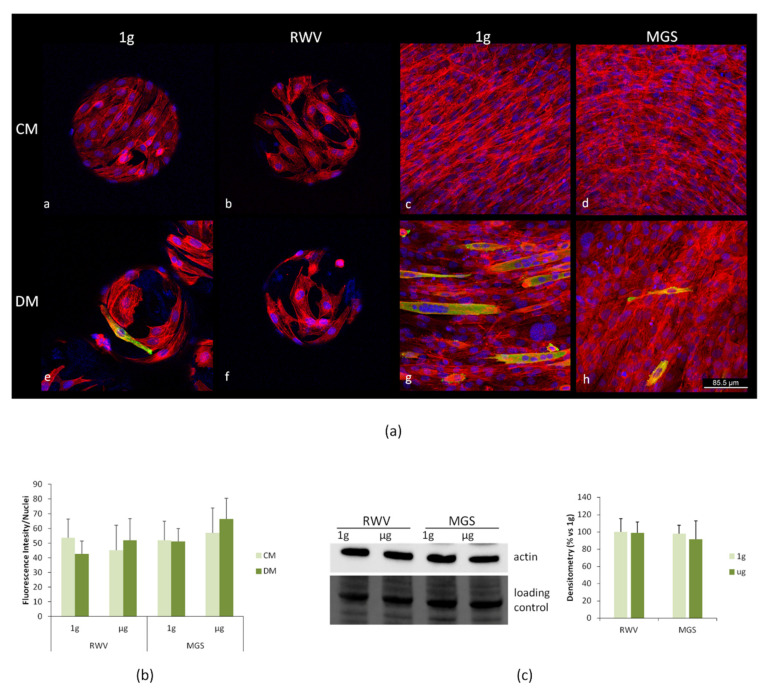
C2C12 were cultured in the RWV and in the MGS or in 1 g-ground control for 4 days. (**a**) The cells were fixed and immunofluorescence was performed using antibodies against myosin heavy chain 3 (MHC). The samples were counterstained with rhodamine labeled phalloidin and 4′,6-diamidine-2′-phenylindole dihydrochloride (DAPI). (**b**) Phalloidin fluorescence was quantified by ImageJ. (**c**) Cell extracts were analyzed for β-actin levels by western blot, and densitometry was performed using ImageJ.

After 4 days in the RVW or in the MGS, C2C12 morphology was examined by confocal microscopy (Figure 6). Important differences occur when the cells are cultured on the beads, which seem to constrain the cells and prevent their elongation. We propose this is the reason why myogenic differentiation in 1g, as evaluated by staining with antibodies against MHC, was markedly delayed in C2C12 cultured on the beads vs. cells in the flasks (Figure 6a, panels e and g). In the latter case, several multinucleated MHC-positive cells could be observed. Multiple nuclei and MHC expression represent a measure of myotubes formation. Simulated microgravity in the RWV changed cell morphology in C2C12 both in CM and DM. The cells were larger and did not elongate. Importantly, we did not find any cell expressing MHC after culture in the RWV with DM (Figure 6a, panel f). On the contrary some cells expressing MHC were observed in the MGS in response to DM, but the cells remained single and thin, which indicates that their differentiation was incomplete (Figure 6a, panel h). The actin fibers were quantified, and no differences were detected (Figure 6b). We then performed a western blot to analyze the total amounts of actin in C2C12 cultured for 4 days in the RWV or the MGS in CM. No significant differences emerged between cells in simulated microgravity and their controls cultured in 1g (Figure 6c).

## 3. Discussion

Cell culture experiments have substantially contributed to many significant breakthroughs in biomedicine, shedding new light on in vivo processes responsible for the formation and function of tissue or organs. Furthermore, pathogenic mechanisms of many diseases have been and still are unveiled thanks to the different approaches developed to culture the cells. 

Space medicine makes no exception. Indeed, spaceflight presents unique medical challenges due to the simultaneous presence of various stressful factors, from space radiation to microgravity, from low physical activity to altered nutritional patterns. Since life blossomed under the constant pull of gravity, microgravity requires adaptive responses to face the dares of such an extreme environment. With the increase in the duration of space missions, it became evident that bone and muscle loss are space-dependent disorders that can be interpreted as adaptive responses to weightlessness [24]. Experiments on cells in real and simulated microgravity have shed some light on the pathogenesis of these disorders. An imbalance between osteoblasts and osteoclasts under gravitational unloading has been found and, in part, explains space-associated osteopenia [25]. Microgravity retards myoblasts proliferation and differentiation, and a recent paper [26] has demonstrated that the dysregulated production of immune-related microRNA has a role in driving muscle atrophy in simulated microgravity.

There is no doubt that more basic science is needed to understand cellular responses to microgravity. However, conducting experiments in space is demanding, and the number of flight opportunities is limited. Thus, the use of alternative tools like bioreactors for benchtop microgravity research represents a valid alternative for designing and validating experiments in real microgravity. It is noteworthy that microgravity-simulating devices are also useful in tissue engineering, since it is possible to expand cell populations and generate 3D tissue structures in a scaffold-free fashion [27]. Consequently, there is a high demand for user-friendly and easily operable microgravity simulators. We developed a novel benchtop device, denominated MGS (microgravity simulator), based on the well-known RPM principle of nullification of the gravity vector via rotation. The MGS design is compact so that it can be accommodated in a commercially available CO_2_ incubator. 

Furthermore, MGS guarantees equal treatment of all the exposed culture flasks, which is not realized by the other available devices. The algorithms controlling the microgravity simulating movements of the MGS have been improved as well. In order to verify the quality of the MGS, we compared the behaviour of myoblasts and bMSC cultured either on the MGS or on widely used microgravity simulators, the RWV and the RPM, respectively. We focused on the effects of simulated microgravity on C2C12 myoblasts and bMSC, because mechanical stimulation is critical for myogenesis and osteogenesis. 

We demonstrated that culture of C2C12 in the MGS and in the RWV downregulated the expression of three markers of myogenesis, i.e., *Myog*, *Mylpf*, and *Myh3*. Our results by confocal microscopy suggest that the culture of C2C12 on beads, as required to use the RWV, impairs or retards C2C12 differentiation, thus indicating that this simulator is not recommended to study myogenic differentiation. Our results are in accordance with previous studies performed in benchtop microgravity simulators [19,28]. Indeed, it has been shown that culture in a 3D clinostat inhibits C2C12 differentiation through the reduced phosphorylation of Akt and, therefore, the inhibition of FOXO1 activation [19]. Similar results were obtained by Calzia et al. [20], who underlined that C2C12 cells in the RPM maintain a non-differentiated phenotype, and this is a fundamental feature for regeneration and transplantation. 

The behavior of bMSC in simulated microgravity is still debated. Some studies have demonstrated that bMSC retain their ability to differentiate into osteoblasts when cultured under simulated microgravity conditions [16,29,30], while others demonstrate that osteogenic differentiation is inhibited [31,32]. Here, we demonstrate that bMSC cultured in MGS and RPM in the presence of an osteogenic cocktail overexpress some osteogenic markers, i.e., *RUNX2*, *Sp7,* and *COL1A1*. Interestingly, culture in the MGS performs better than RPM in inducing bMSC osteogenic response to OM. Importantly, while bMSC in the RPM upregulate *RUNX2* and *Sp7* even in the absence of osteogenic stimuli, this does not happen in the MGS. We hypothesize that RPM generates additional forces or mechanical cues that might trigger unexpected, nonspecific cellular responses. Since bMSC are very sensitive to mechanical stimuli, we suggest MGS as a more appropriate device to study the effects of simulated microgravity in osteogenesis. 

Gravitational forces influence actin cytoskeletal dynamics [33]. In some cell types, cytoskeletal rearrangements are reversible after a few days in simulated microgravity, probably as an adaptive response. For instance, bMSC cultured in the RPM rapidly reorganize their cytoskeleton, and after 4–5 days, the cells regain their actin structures [34]. Similarly, early cytoskeletal alterations were observed in C2C12 in simulated microgravity, but after a few hours the cells seem to adapt, and no major differences are detected [35]. In C2C12 and in bMSC maintained in simulated microgravity for 4 days, we did not observe significant differences in cell shape, actin fiber organization, or the total amounts of actin, in agreement with the aforementioned studies.

Originally created to design experiments in space, various bioreactors, among which the RPM, have been utilized to engineer various tissues such as preliminary vessels, eye tissue, bone, cartilage, and multicellular cancer spheroids. Indeed, weightlessness offers a unique environment for the cells because of its lack of sedimentation and convection. Because of its characteristics, the MGS emerges as a promising tool to study the behaviour of cells cultured in scaffolds as well as of spheroids and organoids, and might become a new device for bioengineering and regenerative medicine.

## 4. Materials and Methods

### 4.1. Description of Algorithm Used to Simulate Microgravity

The basic working principle of microgravity-simulating machines like the RPM is a random positioning of the rotation axis, resulting in an averaged zero-gravity acceleration or apparent force with respect to the cell reference frame. The random positioning can be described as a random walk on the surface of a sphere, changing randomly direction by turning 90° left or right.

After a certain period of time, the path on the sphere shows a homogeneous distribution. This form of probabilistic spatial path distribution can also be calculated and simulated in advance with MATLAB/LabVIEW (MathWorks) [36]. The distribution is measured statistically with the Giné test [37,38,39], which gives a uniformity factor F*. An optimal distribution of the path on the sphere is achieved with the theoretical factor F*_opt_ = 3.633.

**Figure 7:**
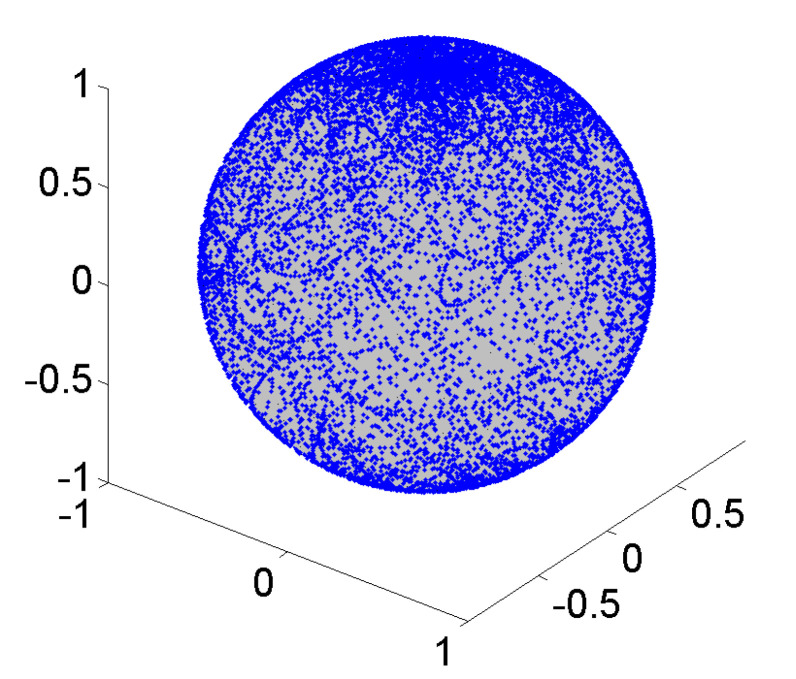
Random walk path distribution on sphere; conventional RPM control schemes lead to concentrations at the poles (low unifomity F* = 188.4).

For operating RPM, the rotating axis has to be controlled by electronic feedback devices. A typical motion and trajectory control algorithm of a random positioning machine will change arbitrarily the direction and velocity of the rotating axis. Randomly controlled rotation will not result in a homogenous path distribution or loading of the cells. While such an algorithm covers the entire rotational space quite uniformly, once wrapped around the unit sphere, concentrations at the poles become apparent, as shown in Figure 7. 

The above mentioned Giné test here gives a significant higher value for the uniformity factor F* = 188.4 (i.e., non-homogenous path distribution).

The crucial criteria for determining the quality of our MGS test-design and planning are as follows:

(1) a uniform path distribution with low uniformity factor F* for zero gravity application (for partial gravity, a controlled concentration of path lines is required)
(2) in-advance (MATLAB) computer simulation of motion path, (not-randomly) control parameters, and statistical measures before the machine is utilized for biological tests.

**Figure 8:**
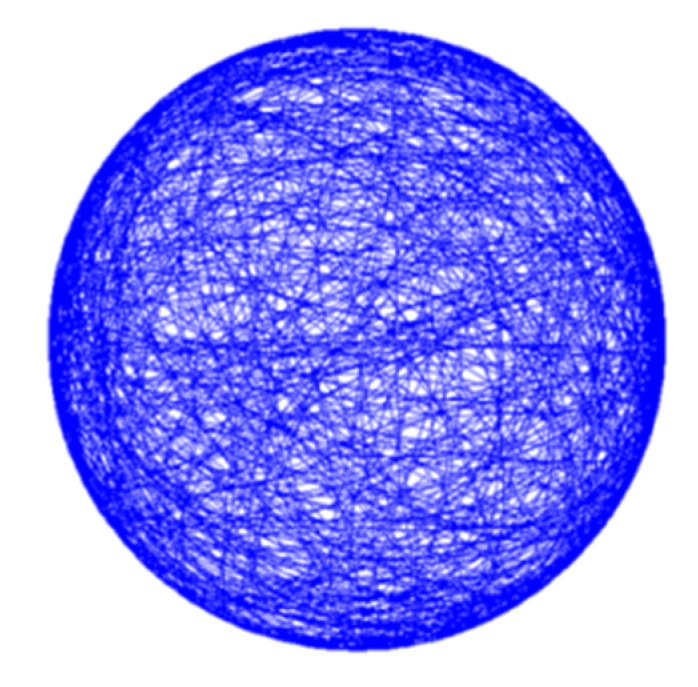
Optimized control scheme leads to homogeneous and controlled path distribution (with excellent uniformity F* = 3.6) for zero or partial gravity applications.

**Figure 9:**
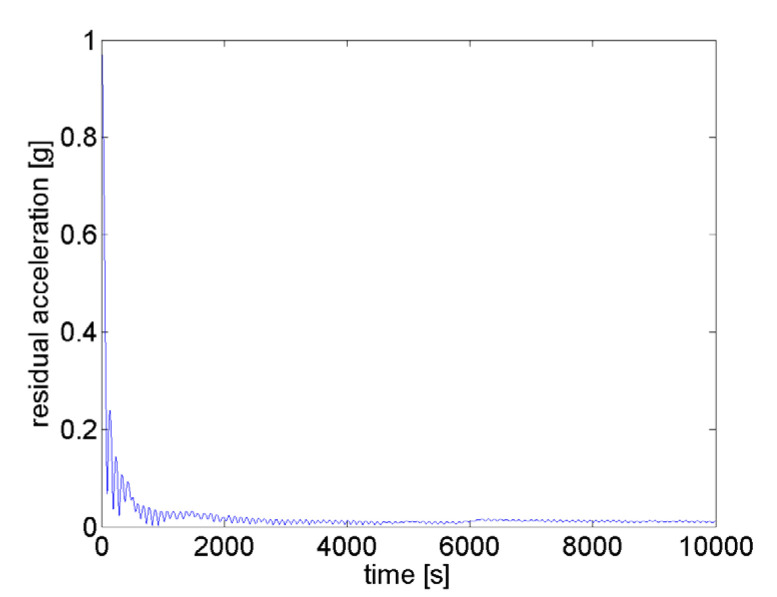
The residual gravity for planned trajectory approaches roughly 0.01 g after a few hundred seconds (nullification).

Figure 8 shows the result for the path distribution on a sphere of the proposed in-advance MATLAB microgravity test design resulting in a significantly lower uniformity factor F* = 3.6. This is realized by an optimized algorithm for trajectory path planning and control, and allows for zero and partial gravity with a minimal uniformity factor F*_opt_ = 3.633. The achieved result for a typical experiment is shown in Figure 9. For zero gravity application, the apparent residual gravity decreases rapidly within a few hundred seconds with final value in the 0.01 g range and below (*μg*).

### 4.2. Cell Culture

Besides validating the MGS mathematically, biological tests were conducted to compare the cellular behaviour on the MGS with the behaviour on the commonly used RPM and RWV. Two different types of cell were used: adherent human bone marrow stem cells (bMSC) and mouse skeletal muscle myoblasts (C2C12). The three bioreactors operated at 37 °C. 1 g-ground control cultures, treated in parallel in identical equipment, were placed on the stationary frame of the RPM, the RWV or the MGS.

For experiments in the RPM or in the MGS, 15 × 10^3^ cells/cm^2^ of bMSC were cultured in flasks completely filled with medium devoid of air bubbles to minimize turbulences and shear stress, and supplemented with 12.5 mM 4-(2-hydroxyethyl)-1-piperazineethanesulfonic acid (HEPES). The cells were used between passage 3 and 5. The flasks were then allocated on the RPM or on the MGS. 

Human bMSC from Lonza (Basilea, Switzerland) were cultured at 37 °C and 5% CO_2_ in Dulbecco’s Modified Eagle’s Medium (DMEM) containing 10% Fetal Bovine Serum (FBS) and 2 mM glutamine (culture medium, CM). All the reagents for cell culture were from Sigma-Aldrich (St. Louis, MO, USA). The cells were used between passage 2 and 5. Osteogenic differentiation was induced in confluent bMSC by adding an osteogenic cocktail containing 10^−8^ M 1α,25-dihydroxyvitamin D_3_ (Vitamin D), 10 mM β-glycerol-phosphate, and 0.05 mM ascorbic acid (osteogenic medium, OM) (Sigma-Aldrich).

C2C12 mouse skeletal myoblasts from the American Type Culture Collection (ATCC) were cultured in DMEM containing 20% FBS. The cells were used between passage 8 and 12. Before being transferred to the RWV, C2C12 were cultured on beads (Cytodex 3, Sigma Aldrich, St. Louis, MO, USA), which were then transferred into the rotating vessels completely filled with medium as reported [23]. For the experiments in the MGS, 25 × 10^3^ cells/cm^2^ were seeded in the flasks, and myogenic differentiation was induced by culturing the cells in 2% horse serum (DM). 

### 4.3. Real-Time PCR

Total RNA was extracted with the PureLink RNA Mini kit (Ambion, Thermo Fisher Scientific, Waltham, MA, USA). Single-stranded cDNA was synthesized from 0.2 µg RNA in a 20 µL final volume using the High Capacity cDNA Reverse Transcription Kit, with RNase inhibitor (Applied Biosystems, Thermo Fisher Scientific) according to the manufacturer’s instructions. Real-time PCR (RT-PCR) was performed three times in triplicate on the CFX96 Touch Real-Time PCR Detection System (Biorad, Hercules, CA, USA) using TaqMan Gene Expression Assays (Life Technologies, Monza, Italy): *RUNX2* (Hs00231692_m1), *Sp7* (Hs01866874_s1), *COL1A1* (Hs00164004_m1), *Myog* (Mm00446194_m1), *Mylpf* (Mm00443940_m1), and *Myh3* (Mm01332463_m1). The housekeeping gene *GAPDH* (Hs99999905_m1, Mm99999915_g1) was used as an internal reference gene. Relative changes in gene expression were analysed by the 2^−ΔΔCt^ method. The experiment was repeated three times in triplicate.

### 4.4. Confocal Imaging

bMSC and C2C12 seeded on flasks or on beads, respectively, were fixed in phosphate buffered saline containing 4% paraformaldehyde and 2% sucrose pH 7.6, permeabilized with Triton 0.3%, incubated with anti-MHC immunopurified IgGs (R&D Systems, Minneapolis, MN, USA) overnight at 4 °C, and stained with an Alexa Fluor 488 secondary antibody (ThermoFisher Scientific). We used rhodamine-labeled phalloidin to visualize the actin filaments of the cytoskeleton, and 4′,6-diamidine-2′-phenylindole dihydrochloride (DAPI, Sigma) was used to stain the nuclei. Finally, cells were mounted with ProLong Gold Antifade Mountant (Invitrogen, Carlsbad, CA, USA), and images were acquired using a 20X objective with a SP8 Leica confocal microscope. The phalloidin fluorescence was quantified by ImageJ software.

### 4.5. Western Blot

bMSC and C2C12 were lysed in 10 mM Tris-HCl (pH 7.4) containing 3 mM MgCl_2_, 10 mM NaCl, 0.1% SDS, 0.1% Triton X-100, 0.5 mM EDTA, and protease inhibitors, separated by SDS-PAGE on Mini-PROTEAN TGX Stain-free Gels (Bio-Rad Laboratories, Hercules, CA, USA) and transferred to nitrocellulose sheets by using Trans-Blot^®^ Turbo™ Transfer Pack (Bio-Rad Laboratories). The immunoblot analysis was performed using primary antibodies against β-actin (Tebu Bio-Santa Cruz, Magenta, Italy). After extensive washing, secondary antibodies labelled with horseradish peroxidase (Amersham Pharmacia Biotech Italia, Cologno Monzese, Italy) were used. Immunoreactive proteins were detected with Clarity™ Western ECL substrate (Bio-Rad Laboratories). The nitrocellulose sheets were used as control loading. The experiments were repeated twice, and a representative blot is shown (Figure 4c or Figure 6c). Densitometry was performed with ImageJ software.

### 4.6. Statistical Analysis

Data are reported as mean ± SD. The data were non-parametric and normally distributed, and were analysed using two-way ANOVA. The *p*-values deriving from multiple pairwise comparisons were corrected using the Bonferroni method. Statistical significance was defined as *p*-value ˂ 0.05. Regarding the Figures, * *p* ˂ 0.05; ** *p* ˂ 0.01; *** *p* ˂ 0.001.

## Abbreviations

MGS	Microgravity simulator
RWV	Rotating Wall Vessel
RPM	Random Positioning Machine
CM	Culture Medium
OM	Osteogenic Medium
RUNX2	Runt-related transcription factor 2
COL1A1	Collagen Type I Alpha 1 Chain
RT-PCR	Real Time-PCR
DM	Myogenic Medium
Myog	Myogenic Factor 4
Mylpf	Myosin Light Chain 2
Myh3	Myosin Heavy Chain 3
MHC	Myosin heavy chain
Akt	Protein Kinase B
FOXO1	Forkhead Box O1
